# Pan-cancer analysis of the *PDE4DIP* gene with potential prognostic and immunotherapeutic values in multiple cancers including acute myeloid leukemia

**DOI:** 10.1515/med-2023-0782

**Published:** 2023-08-28

**Authors:** Qi Li, Yujing Cheng, Wanlu Chen, Ying Wang, Run Dai, Xin Yang

**Affiliations:** Department of Blood Transfusion, The First People’s Hospital of Yunnan Province – The Affiliated Hospital of Kunming University of Science and Technology, 650032 Kunming, Yunnan, China

**Keywords:** *PDE4DIP*, pan-cancer, prognosis, immune, acute myeloid leukemia

## Abstract

Phosphodiesterase 4D interacting protein (*PDE4DIP*) interacts with cAMP-specific phosphodiesterase 4D and its abnormal expression promotes the development of hematological malignancies, breast cancer, and pineal cell carcinoma. However, there is currently no systematic pan-cancer analysis of the association between *PDE4DIP* and various cancers. Thus, this study aimed to elucidate the potential functions of *PDE4DIP* in various cancers. Based on the multiple public databases and online websites, we conducted comprehensive analyses for *PDE4DIP* in various cancers, including differential expression, prognosis, genetic variation, DNA methylation, and immunity. We thoroughly analyzed the specific role of *PDE4DIP* in acute myeloid leukemia (LAML). The results indicated that there were differences in *PDE4DIP* expression in cancers, and in kidney chromophobe, LAML, pheochromocytoma and paraganglioma, thymoma, and uveal melanoma, *PDE4DIP* had potential prognostic value. *PDE4DIP* expression was also correlated with genetic variation, DNA methylation, immune cell infiltration, and immune-related genes in cancers. Functional enrichment analysis showed that *PDE4DIP* was mainly related to immune-related pathways in cancers, and in LAML, *PDE4DIP* was mainly related to immunoglobulin complexes, T-cell receptor complexes, and immune response regulatory signaling pathways. Our study systematically revealed for the first time the potential prognostic and immunotherapeutic value of *PDE4DIP* in various cancers, including LAML.

## 1 Introduction

Phosphodiesterase 4D (*PDE4D*) is an important member of the nucleotide phosphodiesterase superfamily, which can specifically hydrolyze the intracellular second messenger cyclic adenosine monophosphate (cAMP), and plays a vital regulatory role in various activities of cells and the development of malignant tumors [1]. Many studies have pointed out that *PDE4D* is a tumor-promoting molecule in some cancers, including hematological malignancies, lung cancer, prostate cancer, colorectal cancer, gastric cancer, melanoma, head and neck cancer, breast cancer, ovarian cancer, endometrioma, pancreatic ductal adenocarcinoma (PDAC), etc. [1–5]. For example, Rahrmann et al. revealed that *PDE4D* is a common insertion site in prostate cancer, and *PDE4D* knockout can reduce the growth and migration rate of prostate cancer cells *in vitro* [3]. Liu et al. discovered that the up-regulation of *PDE4D* expression in patients with PDAC is closely related to poor prognosis and multiple clinicopathological characteristics, indicating that the *PDE4D* gene may be a potential target for the prognosis and treatment of PDAC [5]. In addition, Dong and Zhang et al. indicated that there are differences in the expression of *PDE4D* in hematological malignancies, i.e., compared to peripheral blood mononuclear cells from healthy adults, the expression of *PDE4D* is significantly reduced in cells extracted from patients with chronic lymphocytic leukemia, while is more abundant in CEM and Jurkat T leukemia cell lines [6,7]. All in all, the above studies demonstrated that *PDE4D* may be a potential target for multiple cancer treatments.

Related studies further found that phosphodiesterase 4D interacting protein (*PDE4DIP*) is a protein that can interact with *PDE4D* and anchor *PDE4D* in the centrosome/Golgi region of cells, and is mainly expressed in the brain and heart [8]. Some studies have shown that there is a close association between *PDE4DIP* gene mutations and atrial fibrillation, stroke, and heart failure [9]. However, there are relatively few reports on the *PDE4DIP* gene and tumor development, only including leukemia, breast cancer, and pineoblastoma. Yao et al. found for the first time in their research on gene mutation profiles in Chinese leukemia patients that high mutations in the *PDE4DIP* gene are significantly associated with the occurrence of leukemia [10]. Onyeisi et al. pointed out that in breast cancer, the most common malignancy in women, the abnormal expression of syndecan-4 is affected by transcriptional and post-transcriptional mechanisms, including *PDE4DIP* gene mutations [11]. Furthermore, Snuderl et al. found that there is a small duplication of the *PDE4DIP* gene in patients with pineal cell tumors and this gene mutation may lead to the overexpression of *PDE4DIP*, which is closely related to the development of pineal cell tumors [12]. However, the full picture of *PDE4DIP* in pan-cancer has not been reported.

In order to fully elucidate the important role of *PDE4DIP* in multiple cancer types, we conducted a systematic pan-cancer analysis of the *PDE4DIP* gene based on The Cancer Genome Atlas (TCGA) [13] and the Genotype-Tissue Expression (GTEx) [14] public databases in this study. We evaluated the differential expression of *PDE4DIP* in various cancers and its relationship with patients’ prognosis, and further analyzed the relevance between *PDE4DIP* expression and genetic variation, DNA methylation, and immune characteristics, as well as the potential biological functions of *PDE4DIP* in pan-cancer. In addition, because previous research has found that *PDE4DIP* is associated with hematological malignancies, we further explored the specific role of *PDE4DIP* in acute myeloid leukemia (LAML) in depth, providing a new target for the future diagnosis and treatment of various cancers, including LAML.

## 2 Methods and materials

### 2.1 Data collection and expression analysis of *PDE4DIP*

The RNA sequencing data (fragments per kilobase million [FPKM]) and clinical data of *PDE4DIP* in 33 cancers were obtained from the TCGA database using UCSC Xena (https://xenabrowser.net/datapages/), and the full names of all cancers are shown in Table S1. The RNA sequencing data (FPKM) of normal tissues were obtained from the GTEx database (https://commonfund.nih.gov/GTEx). During the above process, we used Perl to extract and merge *PDE4DIP* data, and used the R package “limma” to conduct batch correction and differential expression analysis. Then, we used R packages “ggplot2” and “ggpubr” to draw a box diagram of *PDE4DIP* differential expression. Because basal-like breast cancer (BLBC), mesothelioma (MESO), and uveal melanoma (UVM) have no corresponding normal tissues, this diagram did not show them. Afterward, we used the R package “ggradar” to draw a radar map of *PDE4DIP* expression in tumors and healthy tissues, respectively, where the value represents the mean value of *PDE4DIP* expression. Furthermore, based on tumor tissues and their corresponding paracancerous tissues in TCGA data, we used wilcox.test to analyze the differential expression of *PDE4DIP*, in which R packages “ggplot2,” “ggpubr,” and “patchwork” were used for mapping. In addition, based on the CPTAC data set in the UALCAN database (http://ualcan.path.uab.edu/), we explored the differential expression of the *PDE4DIP* protein in tumors and normal tissues.

### 2.2 Prognostic analysis

Based on the Cox regression analysis, we evaluated the relevance between four survival data of tumor patients and *PDE4DIP* expression, including overall survival (OS), disease-specific survival (DSS), disease-free interval (DFI), and progression-free interval (PFI). The R package “survival” was used for the above analysis. Furthermore, we evaluated the relationship between *PDE4DIP* expression and patients’ OS using the Kaplan–Meier (KM) analysis. Specifically, we used Perl to extract expTime.txt survival data and used R packages “survival,” “ggplot2,” “ggpubr,” and “surviver” to analyze and draw survival curves.

### 2.3 Genetic alteration analysis

Gene mutation and copy number variation (CNV) analyses were conducted on the cBioPortal website (http://www.cBioPortal.org/). Pearson’s correlation analysis was used to explore the relationship between *PDE4DIP* expression and CNV in cancers. Correlation analysis was carried out through the R package “corrplot” and the method “spearman,” and R packages “ggplot2” and “ggpubr” were used to draw the lollipop diagram.

### 2.4 DNA methylation analysis

We downloaded the survival evaluation index data (OS, DFI, PFI, and DSS) of cancer patients from the UCSC Xena website (https://xenabrowser.net/datapages/), and matched them with the methylation *β*-values of *PDE4DIP* to further obtain the analysis data. After that, the methylation *β*-values were separated into two groups on the basis of the median value, and R packages “survival,” “ggplot2,” “ggpubr,” and “survivor” were used for drawing KM curves. Pearson’s correlation analysis was used to explore the relationship between *PDE4DIP* expression and DNA methylation in cancers through the R package “corrplot” and the method “spearman.”

### 2.5 Immune level analysis

First, the relevance between *PDE4DIP* expression and tumor-associated macrophage (TAM) infiltration was studied using the TIMER 2.0 online website (http://timer.cistrome.org/), and the heat map was plotted by the R package “pheatmap.” Second, based on the TISIDB website (http://cis.hku.hk/TISIDB/index.phpc), we extracted immune-related gene sets from TCGA, including major histocompatibility complex, chemokine, acceptor, immunoinhibitor, and immunostimulator, and further analyzed their correlation with *PDE4DIP* expression. Then, the above correlation analyses were carried out through the R package “corrplot” and the method “spearman.” Third, based on LAML data in the TCGA database (TCGA-LAML), we used the CIBERSORT algorithm to obtain 22 immune cell scores, and further analyzed their correlation with *PDE4DIP* expression.

### 2.6 Gene set enrichment analysis (GSEA)

The GSEA of *PDE4DIP* in 33 cancers was carried out using R packages “ggplot2,” “limma,” “pheatmap,” “ggsci,” “org.Hs.eg.db,” “patchwork,” and “ggridges.” Afterward, the enrichment results were sorted by the normalized enrichment score (NES) value, and the first 20 pathways were visualized in the form of mountain maps.

### 2.7 Statistical analysis

In this study, all statistical analyses and result maps were completed by R software (v 4.0.2). *p* < 0.05 indicated statistically significant, and we marked the significant results with *, where * represents *p* < 0.05, ** represent *p* < 0.01, *** represent *p* < 0.001.

## 3 Results

### 3.1 Aberrant expression of *PDE4DIP* in pan-cancer

**Figure 1:**
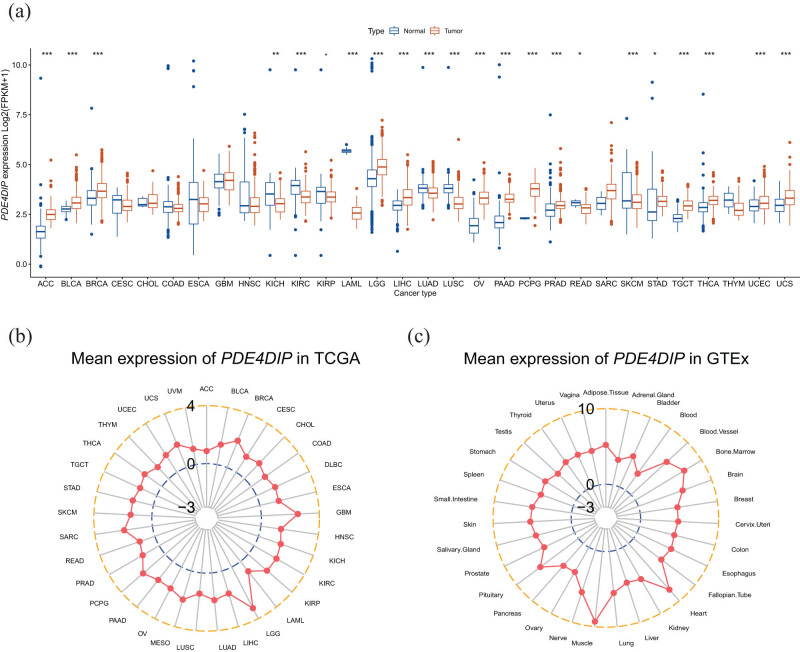
*PDE4DIP* expression pattern at mRNA level: (a) differential expression of *PDE4DIP* in tumor tissues and normal tissues, (b) mean expression of *PDE4DIP* in 33 cancer tissues, and (c) mean expression of *PDE4DIP* in normal tissues. *p < 0.05, **p < 0.01, and ***p < 0.001.

**Figure 2:**
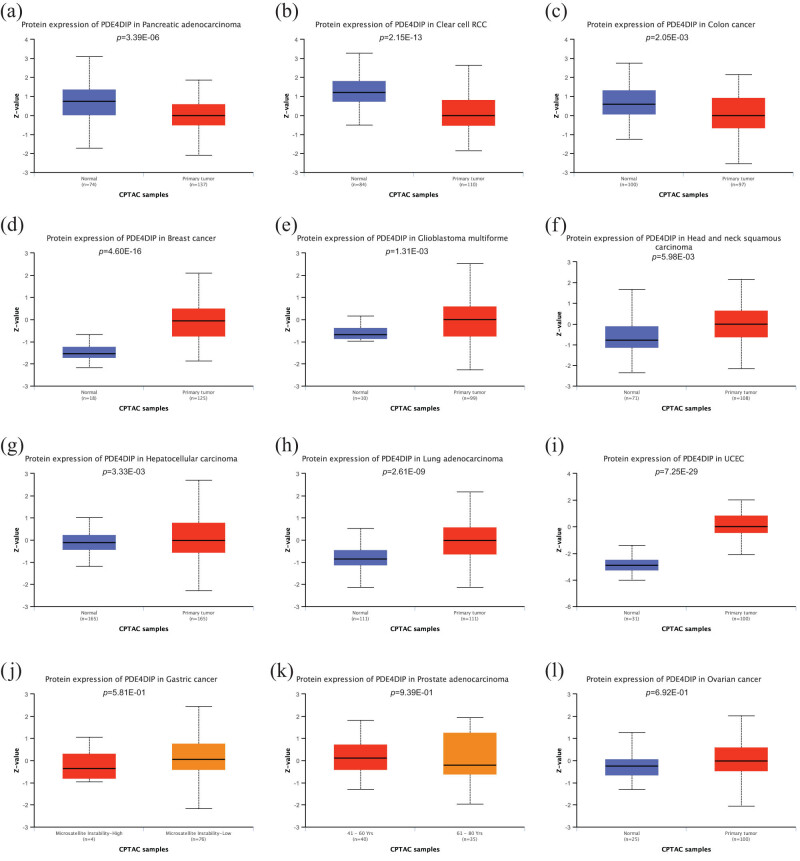
*PDE4DIP* expression pattern in tumor and normal tissues at protein level: (a)–(l) PAAD, KIRC, COAD, BRCA, GBM, HNSC, LIHC, LUAD, UCEC, STAD, PRAD, and OV. *p* < 0.05 indicates statistically significant.

To determine the basic landscape of *PDE4DIP* expression, multi-omics data on *PDE4DIP* levels in various cancers were analyzed. Differential analysis of *PDE4DIP* mRNA expression from the TCGA and GTEx databases revealed that *PDE4DIP* expression was significantly up-regulated in 14 cancers compared to that in healthy tissues, including adrenocortical carcinoma (ACC), bladder urothelial carcinoma (BLCA), breast invasive carcinoma (BRCA), brain lower grade glioma (LGG), liver hepatocellular carcinoma (LIHC), ovarian serous cystadenocarcinoma (OV), pancreatic adenocarcinoma (PAAD), pheochromocytoma and paraganglioma (PCPG), prostate adenocarcinoma (PRAD), stomach adenocarcinoma (STAD), testicular germ cell tumors (TGCT), thyroid carcinoma (THCA), uterine corpus endometrial carcinoma (UCEC), and uterine carcinosarcoma (UCS) (all *p* < 0.05). In contrast, it was significantly down-regulated in eight tumors: kidney chromophobe (KICH), kidney renal clear cell carcinoma (KIRC), kidney renal papillary cell carcinoma (KIRP), LAML, lung adenocarcinoma (LUAD), lung squamous cell carcinoma (LUSC), rectum adenocarcinoma (READ), and skin cutaneous melanoma (SKCM) (all *p* < 0.05) (Figure 1a). The average expression levels of *PDE4DIP* in 33 tumors and healthy tissues are shown in Figure 1b and c. Furthermore, we analyzed the expression of *PDE4DIP* at the protein level in different cancers based on the CPTAC data set. The results showed that the expression of the *PDE4DIP* protein in PAAD, KIRC, and colon adenocarcinoma (COAD) (all *p* < 0.05) was significantly down-regulated compared with that in normal tissues, while it was significantly up-regulated in BRCA, glioblastoma multiforme (GBM), head and neck squamous cell carcinoma (HNSC), LIHC, LUAD, and UCEC (all *p* < 0.05) (Figure 2). The detailed results of the differential expression analysis of *PDE4DIP* at mRNA and protein levels are shown in Tables S2 and S3.

### 3.2 Prognostic value of *PDE4DIP* in pan-cancer

**Figure 3:**
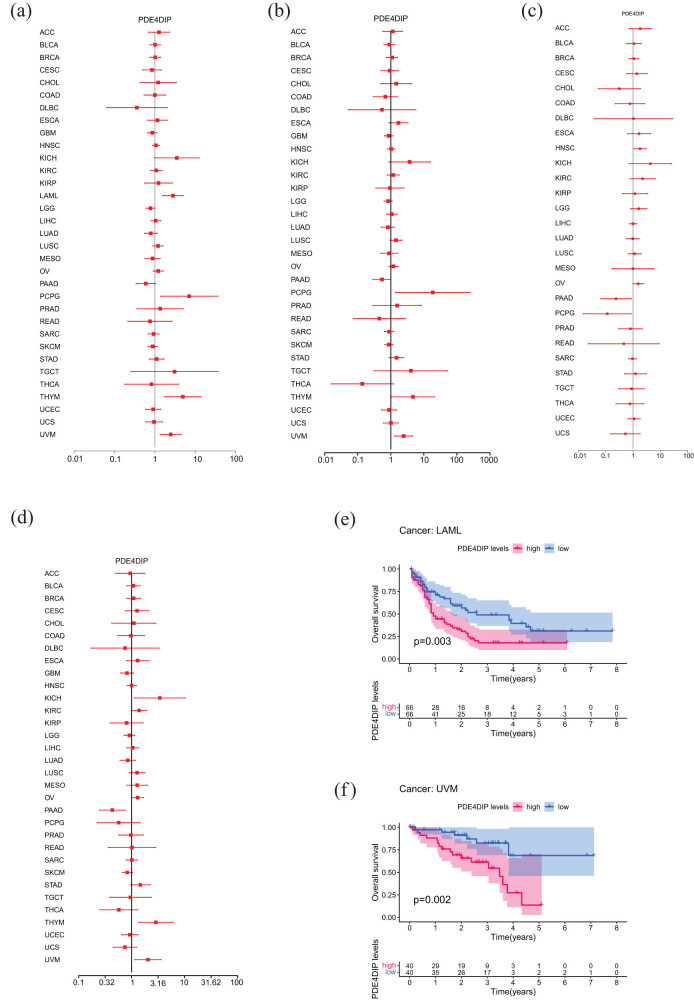
Prognostic analysis of *PDE4DIP* in pan-cancer: (a)–(d) Forest plots of Cox regression analysis for OS, DSS, DFI, and PFI, (e) and (f) KM curves of OS in LAML and UVM. Only significant results are shown and *p* < 0.05 was considered statistically significant.

Prognostic analysis aims to elucidate the association between the expression of a specific gene and the prognosis of cancer patients, thus determining the prognostic value of that gene [15]. Through Cox regression analysis, we studied the relevance between *PDE4DIP* expression and four survival data of cancer patients, including OS, DSS, DFI, and PFI, to further reveal the potential prognostic value of *PDE4DIP* in different cancers. The results of the Cox proportional hazards model showed that the expression of *PDE4DIP* was significantly correlated with lower OS in patients with LAML (*p* = 0.001), PCPG (*p* = 0.020), thymoma (THYM) (*p* = 0.002), and UVM (*p* = 0.003) (Figure 3a). Univariate Cox regression analysis showed that high *PDE4DIP* expression was associated with a significant decrease in DSS in PCPG (*p* = 0.028), THYM (*p* = 0.043), and UVM (*p* = 0.006) patients (Figure 3b), as well as poor DFI in HNSC (*p* = 0.023) patients, while it was significantly associated with better DFI in PAAD (*p* = 0.033) and PCPG (*p* = 0.034) patients (Figure 3c). For PFI, high *PDE4DIP* expression was associated with the significant reduction in PFI in KICH (*p* = 0.030), THYM (*p* = 0.006), and UVM (*p* = 0.016) patients, while it was significantly associated with better PFI in PAAD (*p* = 0.003) patients (Figure 3d). In addition, KM curves further showed that the high expression of *PDE4DIP* was significantly related to the shortened survival time of LAML (*p* = 0.003) and UVM (*p* = 0.002) (Figure 3e and f) patients, indicating that *PDE4DIP* had a poor prognosis in LAML and UVM patients.

### 3.3 Association of *PDE4DIP* expression with genetic alterations

Alterations in specific gene sequences or DNA copy numbers can potentially lead to changes in gene expression or abnormalities in gene function [16]. In cancer-related studies, evaluating the genetic variation profiles of a specific gene or exploring the relationship between these variations and gene expression levels can further determine whether specific alterations within that gene are associated with tumor occurrence, progression, and treatment response [17–19]. Genetic variation analysis pointed out that the mutation frequency of *PDE4DIP* was higher in endometrial cancer, bladder cancer, melanoma, non-small cell lung cancer (NSCLC), hepatobiliary cancer, and BRCA, and the main types included mutation and amplification (Figure S1). CNV indicated that *PDE4DIP* expression was positively related to DNA copy number in most cancers, including UCEC, sarcoma (SARC), KICH, BRCA, BLCA, esophageal carcinoma (ESCA), READ, OV, THCA, KIRC, GBM, COAD, LUAD, STAD, KIRP, cervical squamous cell carcinoma and endocervical adenocarcinoma, LIHC, LUSC, SKCM, and PRAD (Figure S2a).

### 3.4 Association of *PDE4DIP* expression with DNA methylation

**Figure 4:**
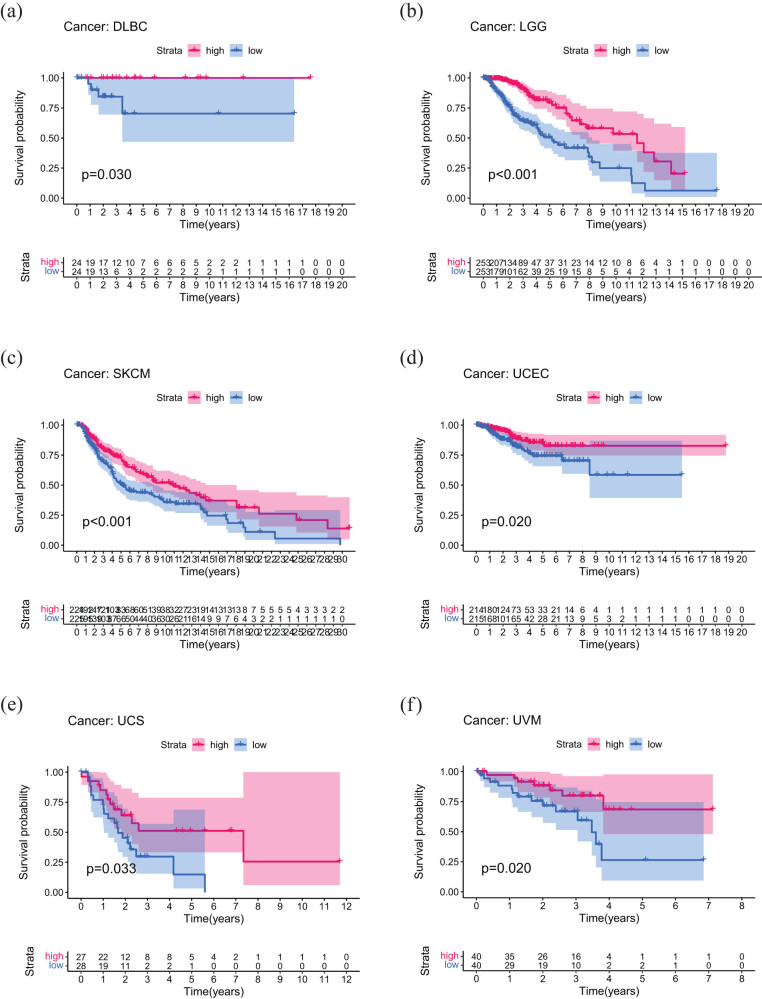
Association of *PDE4DIP* methylation with OS in DLBC, LGG, SKCM, UCEC, UCS, and UVM: (a)–(f) only significant results are shown.

DNA methylation is an epigenetic modification, which is closely related to gene expression and regulation [20]. During cancer progression, abnormal DNA methylation patterns are often accompanied, which will result in abnormal expression of some genes and disorder of normal cell functions, thus promoting the development of tumors [21,22]. DNA methylation analysis showed that in GBM, COAD, LUSC, PAAD, HNSC, LGG, UCEC, and BLCA patients, *PDE4DIP* expression was significantly positively related to DNA methylation, while was significantly negatively related to DNA methylation in STAD, KIRC, SARC, MESO, THCA, THYM, PCPG, and UVM patients (Figure S2b). Furthermore, we evaluated the prognostic value of *PDE4DIP* methylation based on OS, DSS, DFI, and PFI. The results showed that *PDE4DIP* methylation was a prognostic factor for OS of KIRC, PAAD, LGG, SKCM, UCEC, and UVM patients. To be specific, the high level of *PDE4DIP* methylation was significantly related to the increase of OS in patients with PAAD, LGG, SKCM, UCEC, and UVM (Figure 4b–f), but only to the decrease of OS in patients with KIRC (Figure 4a). In addition, *PDE4DIP* methylation was a prognostic factor for DSS of DLBC, LGG, SKCM, UCEC, UCS, and UVM patients (Figure S3), and for PFI in patients with LGG, MESO, UVM, UCEC, UCS, and ACC (Figure S4). However, there was no significant correlation between *PDE4DIP* methylation and DFI in any cancers.

### 3.5 Association of *PDE4DIP* expression with immune-related characteristics

**Figure 5:**
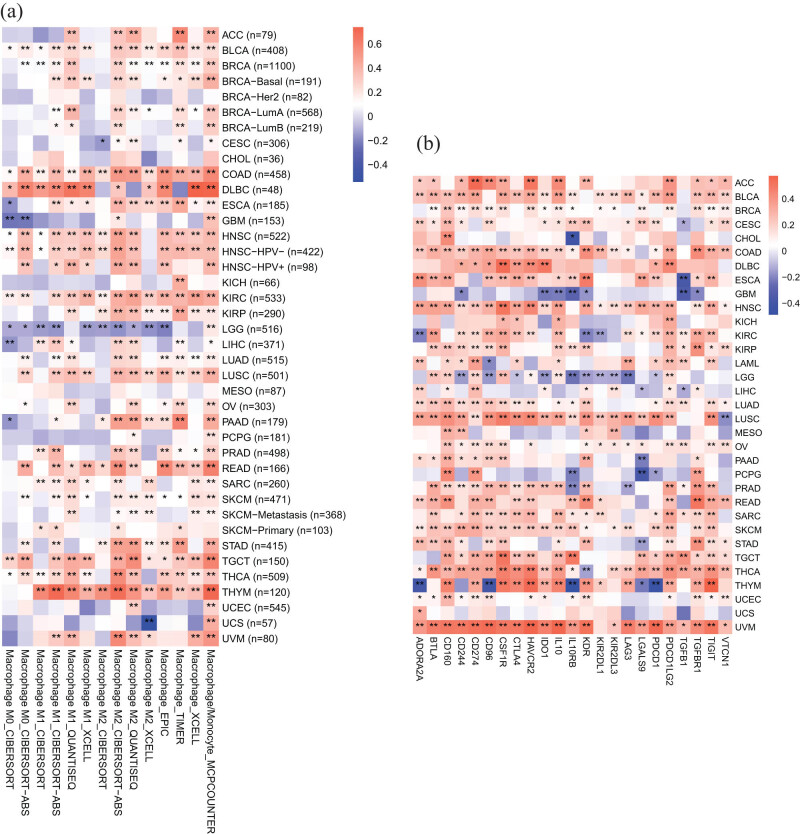
Correlation between *PDE4DIP* expression and immune cell infiltration: (a) correlation between *PDE4DIP* expression and macrophages in pan-cancer and (b) correlation between *PDE4DIP* expression and immunosuppressive factors in pan-cancer. *p < 0.05, **p < 0.01, and ***p < 0.001.

Tumor-infiltrating immune cells are typically dysfunctional, fail to control tumor growth and may even promote tumor development and immune escape [23]. Moreover, abnormal expression of immune-related genes can lead to reduced antigen presentation, and increased production of immunosuppressive substances, and tumor immune escape, thereby impacting tumor immune therapy [24]. Based on the TIMER 2.0 online website, we studied the relevance between TAM infiltration and *PDE4DIP* expression in the tumor microenvironment (TME). The results showed that there was a significant positive correlation between TAM infiltration levels and *PDE4DIP* expression in most cancers, mainly including M2-like TAMs (Figure 5a). Furthermore, based on the TISIDB online website, we further evaluated the potential relevance between immune-related genes and *PDE4DIP* expression in the TME. Notably, gene co-expression analysis revealed that *PDE4DIP* expression was significantly positively related to the expression of most immunoinhibitors in cancers (Figure 5b; Figure S5a–d).

### 3.6 Biological function of *PDE4DIP* in pan-cancer

GSEA of single gene determined the pathways affected by *PDE4DIP* expression in pan-cancer. According to the ranking of the NES value, the first 20 pathways were markedly enriched in 33 kinds of cancers. Among them, the pathways that appeared more than ten times mainly included tuberculosis, Epstein-Barr virus infection, JAK-STAT signaling pathway, chemokine signaling pathway, NOD-like receptor signaling pathway, Th17 cell differentiation, cell adhesion molecules, etc.

### 3.7 Role of *PDE4DIP* in LAML

**Figure 6:**
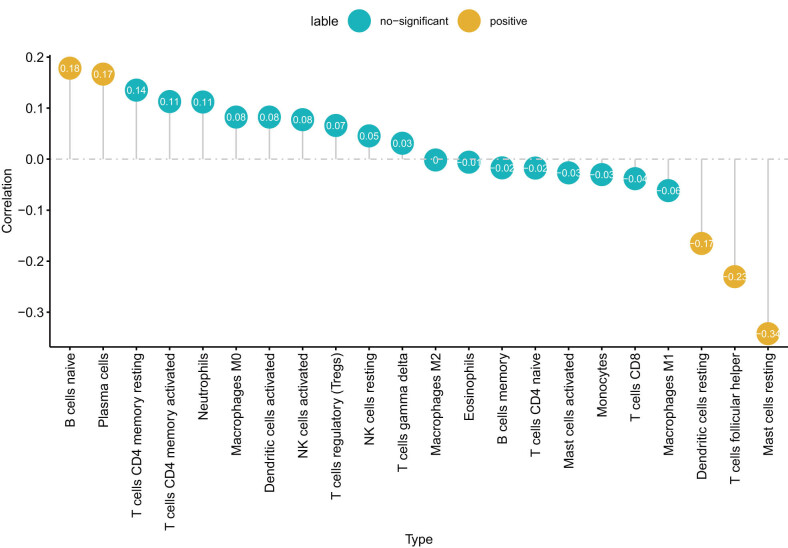
Correlation between *PDE4DIP* expression and immune cell infiltration in LAML.

Based on the TCGA-LAML data, we analyzed the correlation between *PDE4DIP* expression and immune cell infiltration in LAML. The results revealed that *PDE4DIP* expression was significantly positively related to the infiltration of B cell naive and plasma cells, but negatively related to the infiltration of dendritic cell resting, T cell follicular helper, and mast cell resting (Figure 6).

**Figure 7:**
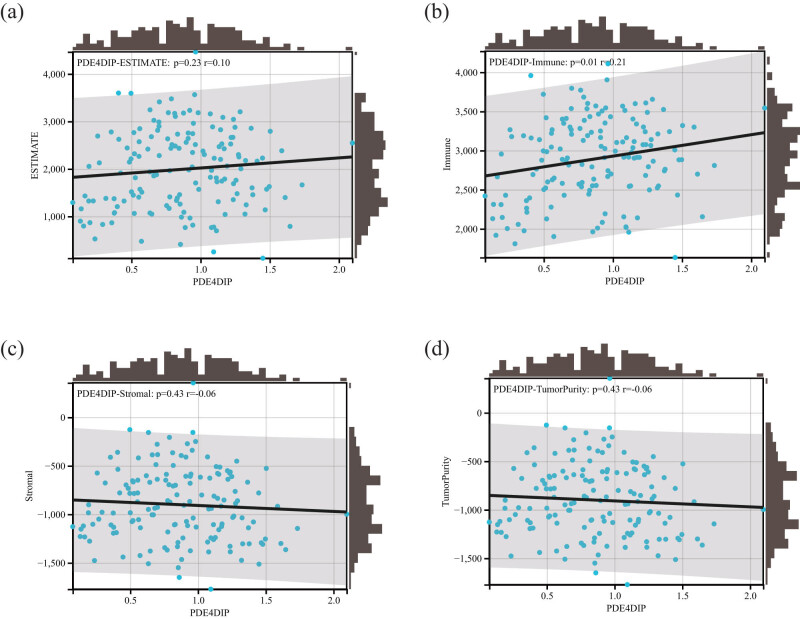
Correlation matrixes between *PDE4DIP* gene expression and (a) estimate score, (b) immune score, (c) stromal score, and (d) TumorPurity score in LAML.

In order to better clarify the role of *PDE4DIP* in LAML and its relationship with immune response, TME, and tumor purity, we conducted correlation analyses between *PDE4DIP* expression and estimate score, immune score, normal score, and TumorPurity score. The results revealed that there was a significant positive association between *PDE4DIP* expression and immune score, indicating that the *PDE4DIP* gene might have a certain impact on the immune response process of LAML, but there was no significant correlation between the *PDE4DIP* gene and estimate score, normal score, and TumorPurity score (Figure 7a–d).

**Figure 8:**
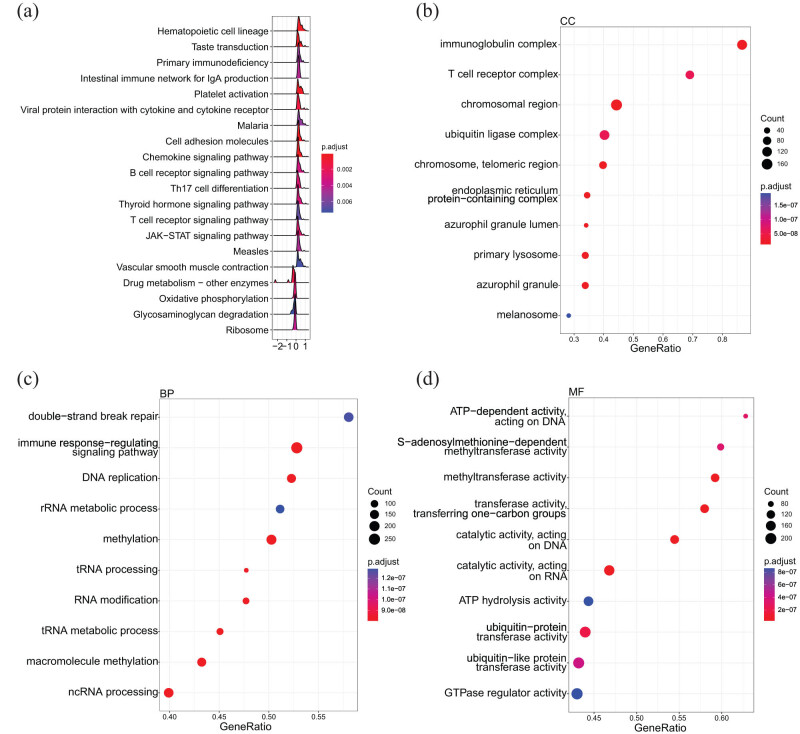
Enrichment analysis of *PDE4DIP* in LAML: (a) GSEA, (b) GO enrichment analysis about cellular component, (c) biological process, and (d) molecular function.

We further explored the potential function of *PDE4DIP* in LAML. GSEA showed that *PDE4DIP* was most markedly enriched in immune-related pathways in LAML, including primary immunodeficiency, intestinal immune network for IgA production, viral protein interaction with cytokine and cytokine receptor, cell adhesion molecules, chemokine signaling pathway, B cell receptor signaling pathway, Th17 cell differentiation, T cell receptor signaling pathway, and JAK-STAT signaling pathway (Figure 8a). Gene ontology (GO) functional enrichment analysis revealed that in LAML, *PDE4DIP* was mainly enriched in immunoglobulin complex, T cell receptor complex, chromosomal region, ATP-dependent activity acting on DNA, methyltransferase activity, double-strand break repair, immune response-regulating signaling pathway, etc. (Figure 8b–d).

## 4 Discussion

As we all know, *PDE4DIP* is a protein that can interact with cAMP-specific *PDE4D* and is widely expressed in various cell types, including neurons, cardiomyocytes, immune cells, and tumor cells, and participates in cell proliferation, migration, and cell cycle [8,25,26]. Previous studies have found that *PDE4DIP* exhibits abnormal expression in various diseases, including malignant tumors [9–12], but its effect on the diagnosis, prognosis, and TME of tumors remains to be further explored. Therefore, our study is the first to comprehensively and systematically analyze the expression, prognosis, genetic changes, DNA methylation, immune cell infiltration, immune-related genes, and biological functions of *PDE4DIP* in many cancers. Our research revealed that the *PDE4DIP* gene had potential prognostic and immunotherapeutic values in various cancers, including LAML, providing a certain theoretical basis for using *PDE4DIP* as a biomarker in future cancer treatment.

Previous studies have pointed out that different tumors exhibit tumor heterogeneity due to genetic and epigenetic changes in the evolution and development of tumors [27]. Consistent with previous reports, our study found that *PDE4DIP* expression was heterogeneous in different cancers, where it was up-regulated in some cancer types and down-regulated in others. Although the exact mechanisms remain unclear, multiple factors could result in the differential expression of *PDE4DIP*. On the one hand, differences in gene expression regulatory networks between different cancer types might result in different regulatory mechanisms of *PDE4DIP*, leading to different expression patterns in cancers [10–12]. On the other hand, *PDE4DIP* expression can be influenced by various factors, including transcription factor regulation, epigenetic modifications, and activation of signaling pathways. And relevant studies have revealed that the *PDE4DIP* expression may be related to some signaling pathways, such as PI3K/AKT, RAS/ERK, and NF1/RAS pathways [28,29]. However, the specific regulatory mechanism of *PDE4DIP* expression in different cancers needs further exploration in subsequent studies. It is worth noting that *PDE4DIP* was significantly up-regulated in many cancer types, including ACC, BLCA, BRCA, LGG, LIHC, OV, PAAD, PCPG, PRAD, STAD, TGCT, THCA, UCEC, and UCS. It is speculated that *PDE4DIP* may serve as an oncogene in these cancers, possibly promoting tumor development. In addition, we compared the expression patterns of *PDE4DIP* at the mRNA and protein levels (Table S4), and the results showed that in some cancer types, the expression patterns of *PDE4DIP* at the mRNA and protein levels were inconsistent, such as in PAAD and LUAD. We speculated that this may be related to the changes in post-transcriptional regulation [30], the differences in post-translational modification [31], and the differences in sample sources. In future research, we will collect more clinical data or design relevant experiments to further clarify the expression pattern of *PDE4DIP* in different cancers.

Prognostic analysis based on OS, DSS, DFI, and PFI indicated that high expression of *PDE4DIP* had a potential prognostic effect on some cancers, with a poor prognosis in LAML, PCPG, THYM, UVM, and HNSC patients, and a good prognosis in PAAD and PCPG patients. Previous studies have reported that the treatment of LAML remained unchanged for most of the twentieth and early twenty-first centuries, and the survival curve remained stagnant for decades [32]. UVM is one of the most invasive and deadly skin cancers, and its mortality increases with the increase of incidence rate [33]. Immunotherapy and targeted therapy can markedly improve the survival rate of patients, but due to the complexity of cancer treatment, seeking the best treatment still requires continuous efforts. In this study, we found that *PDE4DIP* had potential prognostic value in pan-cancer, including LAML and UVM, giving a new guidance for the diagnosis and treatment of cancers in the future.

*PDE4DIP* is an anchor protein, and its genetic mutations can result in impaired cell function, further leading to changes in intracellular targeting and cell survival, and the occurrence of diseases such as atrial fibrillation, stroke, and heart failure [9,34]. In this study, we further found that *PDE4DIP* had a higher frequency of mutations in some cancers, including endometrial cancer, bladder cancer, melanoma, NSCLC, hematological malignancies, and BRCA. Chang et al. conducted whole-exome or targeted gene sequencing of tumor regions in patients with NSCLC and found that many gene mutations occur in patients’ bodies, and certain gene-specific mutation patterns may affect targeted treatment of cancers [35]. Onyeisi et al. found that mutations in the *PDE4DIP* gene may be a key factor leading to the imbalance of expression of cell surface heparan sulfate proteoglycan syndecan-4, thereby promoting the development of BRCA [11]. In addition, Yao et al. found that the *PDE4DIP* gene is one of the highly mutated genes during the occurrence and development of leukemia [10]. All in all, given the high mutation frequency of *PDE4DIP* in various cancers, we speculated that *PDE4DIP* mutations might be closely related to the development of cancers, which needs to be verified through subsequent experiments in different cancers.

Numerous studies have pointed out that TME has a notable effect on the progression of tumors, including immune cells, blood vessels, fibroblasts, lymphocytes, endothelial cells, and extracellular components (cytokines, hormones, etc.) [36,37]. The content of various immune cell infiltrating tumor tissues and the expression level of immune-related genes are closely related to the immune regulation and prognosis of patients [38]. Our study revealed that *PDE4DIP* expression was markedly correlated with immune cell infiltration and the expression of immune-related genes in various cancers. In LAML, *PDE4DIP* expression was positively related to B cell naive and plasma cell infiltration, and negatively related to dendritic cell resting, T cell follicular helper, and mast cell resting infiltration. Moreover, TME score showed that there was a significant positive association between *PDE4DIP* expression and immune score in AML. These results indicate that *PDE4DIP* plays a vital role in the possible immune regulation of tumors, which may become a new marker for cancer immunotherapy.

Finally, through functional enrichment analysis, we found that *PDE4DIP* had a critical impact on the immunotherapy of various cancers, i.e., *PDE4DIP* expression was mainly associated with immune-related pathways such as JAK-STAT signaling pathway, chemokine signaling pathway, and NOD-like receptor signaling pathway, and in LAML, *PDE4DIP* expression was also related to immune modulators such as immunoglobulin complexes and T-cell receptor complexes. Currently, immunotherapy is regarded as a promising option for cancer treatment [39]. Our findings further provide more theoretical basis for using *PDE4DIP* as a biomarker for future immunotherapy of various cancers.

Inevitably, there are some shortcomings in our research that are worth considering. Our research mainly studied the potential role of *PDE4DIP* expression in multiple cancer types based on online public databases, which may have systematic errors and lack of large-scale clinical cohort data for further analysis and verification. At the same time, the specific role of *PDE4DIP* in cancers, especially in LAML, still needs to be further clarified through molecular experiments.

## 5 Conclusion

In summary, our study first conducted a comprehensive analysis of the *PDE4DIP* gene in pan-cancer, revealing its potential prognostic and immunotherapeutic values in various cancers, including LAML, and providing a theoretical basis for the future use of the *PDE4DIP* gene as a potential target for the treatment of cancers.

## Abbreviations

ACC: adrenocortical carcinoma
LAML: acute myeloid leukemia
LGG: brain lower grade glioma
BLBC: basal-like breast cancer
BLCA: bladder urothelial carcinoma
BRCA: breast invasive carcinoma
CHOL: cholangiocarcinoma
COAD: colon adenocarcinoma
CNV: copy number variation
CLL: chronic lymphocytic leukemia
DSS: disease-specific survival
DFI: disease-free interval
ESCA: esophageal carcinoma
GTEx: genotype-tissue expression
GSEA: gene set enrichment analysis
GO: gene ontology
GBM: glioblastoma multiforme
HNSC: head and neck squamous cell carcinoma
KM: Kaplan–Meier
KICH: kidney chromophobe
KIRC: kidney renal clear cell carcinoma
KIRP: kidney renal papillary cell carcinoma
LIHC: liver hepatocellular carcinoma
LUAD: lung adenocarcinoma
LUSC: lung squamous cell carcinoma
MHC: major histocompatibility complex
MESO: mesothelioma
NES: Normalized Enrichment Score
OV: ovarian serous cystadenocarcinoma
OS: overall survival
PDE4DIP: phosphodiesterase 4D interacting protein
PDE4D: phosphodiesterase 4D
PFI: progression-free interval
PDAC: pancreatic ductal adenocarcinoma
PAAD: pancreatic adenocarcinoma
PCPG0: prostate adenocarcinoma
READ: rectum adenocarcinoma
SARC: sarcoma
SKCM: skin cutaneous melanoma
STAD: stomach adenocarcinoma
TCGA: The Cancer Genome Atlas
TIME: tumor immune microenvironment
TAMs: tumor-associated macrophages
TGCT: testicular germ cell tumors
THCA: thyroid carcinoma
THYM: thymoma
UCEC: uterine corpus endometrial carcinoma
UCS: uterine carcinosarcoma
UVM: uveal melanoma

## Supplementary Material

Supplementary material

## References

[1] Hsien Lai S, Zervoudakis G, Chou J, Gurney ME, Quesnelle KM. PDE4 subtypes in cancer. Oncogene. 2020;39(19):3791–802. 10.1038/s41388-020-1258-8. PMC7444459 32203163

[2] Pullamsetti SS, Banat GA, Schmall A, Szibor M, Pomagruk D, Hanze J, et al. Phosphodiesterase-4 promotes proliferation and angiogenesis of lung cancer by crosstalk with HIF. Oncogene. 2013;32(9):1121–34. 10.1038/onc.2012.136. 22525277

[3] Rahrmann EP, Collier LS, Knutson TP, Doyal ME, Kuslak SL, Green LE, et al. Identification of PDE4D as a proliferation promoting factor in prostate cancer using a Sleeping Beauty transposon-based somatic mutagenesis screen. Cancer Res. 2009;69(10):4388–97. 10.1158/0008-5472.CAN-08-3901. PMC2710962 19401450

[4] Cao B, Wang K, Liao JM, Zhou X, Liao P, Zeng SX, et al. Inactivation of oncogenic cAMP-specific phosphodiesterase 4D by miR-139-5p in response to p53 activation. Elife. 2016;5:e15978. 10.7554/eLife.15978. PMC4959878 27383270

[5] Liu F, Ma J, Wang K, Li Z, Jiang Q, Chen H, et al. High expression of PDE4D correlates with poor prognosis and clinical progression in pancreaticductal adenocarcinoma. J Cancer. 2019;10(25):6252–60. 10.7150/jca.35443. PMC6856734 31772658

[6] Dong H, Zitt C, Auriga C, Hatzelmann A, Epstein PM. Inhibition of PDE3, PDE4 and PDE7 potentiates glucocorticoid-induced apoptosis and overcomes glucocorticoid resistance in CEM T leukemic cells. Biochem Pharmacol. 2010;79(3):321–9. 10.1016/j.bcp.2009.09.001. 19737543

[7] Zhang L, Murray F, Zahno A, Kanter JR, Chou D, Suda R, et al. Cyclic nucleotide phosphodiesterase profiling reveals increased expression of phosphodiesterase 7B in chronic lymphocytic leukemia. Proc Natl Acad Sci U S A. 2008;105;(49):19532–37. 10.1073/pnas.0806152105. PMC2614795 19033455

[8] Shapshak P. Molecule of the month, PDE4DIP. Bioinformation. 2012;8(16):740–1. 10.6026/97320630008740. PMC3449385 23055623

[9] Mani A. PDE4DIP in health and diseases. Cell Signal. 2022;94:110322. 10.1016/j.cellsig.2022.110322. PMC9618167 35346821

[10] Yao H, Wu C, Chen Y, Guo L, Chen W, Pan Y, et al. Spectrum of gene mutations identified by targeted next-generation sequencing in Chinese leukemia patients. Mol Genetics Genomic Med. 2020;8(9):e1369. 10.1002/mgg3.1369. PMC7507579 32638549

[11] Onyeisi JOS, Lopes CC, Götte M. Role of syndecan-4 in breast cancer pathophysiology. Am J Physiol-Cell Physiol. 2022;323(5):C1345–54. 10.1152/ajpcell.00152.2022. 36094435

[12] Snuderl M, Kannan K, Pfaff E, Wang S, Stafford JM, Serrano J, et al. Recurrent homozygous deletion of DROSHA and microduplication of PDE4DIP in pineoblastoma. Nat Commun. 2018;9(1):2868. 10.1038/s41467-018-05029-3. PMC6054684 30030436

[13] Wang Z, Jensen MA, Zenklusen JC. A practical guide to The Cancer Genome Atlas (TCGA). Methods Mol Biol. 2016;1418:111–41. 10.1007/978-1-4939-3578-9_6. 27008012

[14] Lonsdale J, Thomas J, Salvatore M, Phillips R, Lo E, Shad S, et al. The Genotype-Tissue Expression (GTEx) project. Nat Genet. 2013;45(6):580–5. 10.1038/ng.2653. PMC4010069 23715323

[15] Moradi Binabaj M, Bahrami A, Khazaei M, Ryzhikov M, Ferns GA, Avan A, et al. The prognostic value of cyclin D1 expression in the survival of cancer patients: a meta-analysis. Gene. 2020;728:144283. 10.1016/j.gene.2019.144283. 31838249

[16] Alzu’bi AA, Zhou L, Watzlaf VJM. Genetic variations and precision medicine. Perspect Health Inf Manag. 2019;16(Spring):1a PMC6462879 31019429

[17] Park JH, Jeong GH, Lee KS, Lee KH, Suh JS, Eisenhut M, et al. Genetic variations in MicroRNA genes and cancer risk: a field synopsis and meta-analysis. Eur J Clin Invest. 2020;50(4):e13203. 10.1111/eci.13203. 31984489

[18] D’Alicandro V, Romania P, Melaiu O, Fruci D. Role of genetic variations on MHC class I antigen-processing genes in human cancer and viral-mediated diseases. Mol Immunol. 2019;113:11–5. 10.1016/j.molimm.2018.03.024. 29625843

[19] Sun H, Deng Q, Pan Y, He B, Ying H, Chen J, et al. Association between estrogen receptor 1 (ESR1) genetic variations and cancer risk: a meta-analysis. J Buon. 2015;20(1):296–308. 25778331

[20] Ehrlich M. Expression of various genes is controlled by DNA methylation during mammalian development. J Cell Biochem. 2003;88(5):899–910. 10.1002/jcb.10464. 12616529

[21] Mahmoud AM, Ali MM. Methyl donor micronutrients that modify DNA methylation and cancer outcome. Nutrients. 2019;11(3):608. 10.3390/nu11030608. PMC6471069 30871166

[22] Holčáková J. Effect of DNA methylation on the development of cancer. Klinicka Onkol. 2018;31(Suppl 2):41–5. 10.14735/amko20182S41. 31023023

[23] Zhong A, Chen T, Xing Y, Pan X, Shi M. FUCA2 is a prognostic biomarker and correlated with an immunosuppressive microenvironment in pan-cancer. Front Immunol. 2021;12:758648. 10.3389/fimmu.2021.758648. PMC8565374 34745134

[24] Zhu R, Tao H, Lin W, Tang L, Hu Y. Identification of an immune-related gene signature based on immunogenomic landscape analysis to predict the prognosis of adult acute myeloid leukemia patients. Front Oncol. 2020;10:574939. 10.3389/fonc.2020.574939. PMC7714942 33330048

[25] Mohamed BA, Elkenani M, Mobarak S, Marques Rodrigues D, Annamalai K, Schnelle M, et al. Hemodynamic stress-induced cardiac remodelling is not modulated by ablation of phosphodiesterase 4D interacting protein. J Cell Mol Med. 2022;26(16):4440–52. 10.1111/jcmm.17468. PMC9357604 35860864

[26] Du Z, Wu B, Xia Q, Zhao Y, Lin L, Cai Z, et al. LCN2-interacting proteins and their expression patterns in brain tumors. Brain Res. 2019;1720:146304. 10.1016/j.brainres.2019.146304. 31233712

[27] Pe’er D, Ogawa S, Elhanani O, Keren L, Oliver TG, Wedge D. Tumor heterogeneity. Cancer Cell. 2021;39(8):1015–7. 10.1016/j.ccell.2021.07.009. 34375606

[28] Potashkin JA, Bottero V, Santiago JA, Quinn JP. Bioinformatic analysis reveals phosphodiesterase 4D-interacting protein as a key frontal cortex dementia switch gene. Int J Mol Sci. 2020;21(11):3787. 10.3390/ijms21113787. PMC7313474 32471155

[29] Pan R, Dai J, Liang W, Wang H, Ye L, Ye S, et al. PDE4DIP contributes to colorectal cancer growth and chemoresistance through modulation of the NF1/RAS signaling axis. Cell Death Dis. 2023;14(6):373. 10.1038/s41419-023-05885-y. PMC10290635 37355626

[30] Zhang JG, Xu C, Zhang L, Zhu W, Shen H, Deng HW. Identify gene expression pattern change at transcriptional and post-transcriptional levels. Transcription. 2019;10(3):137–46. 10.1080/21541264.2019.1575159. PMC6602563 30696368

[31] Han ZJ, Feng YH, Gu BH, Li YM, Chen H. The post-translational modification, SUMOylation, and cancer review. Int J Oncol. 2018;52(4):1081–94. 10.3892/ijo.2018.4280. PMC5843405 29484374

[32] Pelcovits A, Niroula R. Acute myeloid leukemia: a review. Rhode Isl Med J. 2020;103(3):38–40. 32236160

[33] Teixido C, Castillo P, Martinez-Vila C, Arance A, Alos L. Molecular markers and targets in melanoma. Cells. 2021;10(9):2320. 10.3390/cells10092320. PMC8469294 34571969

[34] Abou Ziki MD, Bhat N, Neogi A, Driscoll TP, Ugwu N, Liu Y, et al. Epistatic interaction of PDE4DIP and DES mutations in familial atrial fibrillation with slow conduction. Hum Mutat. 2021;42(10):1279–93. 10.1002/humu.24265. PMC8434967 34289528

[35] Chang YS, Tu SJ, Chen YC, Liu TY, Lee YT, Yen JC, et al. Mutation profile of non-small cell lung cancer revealed by next generation sequencing. Respiratory Res. 2021;22(1):3. 10.1186/s12931-020-01608-5. PMC7789556 33407425

[36] Binnewies M, Roberts EW, Kersten K, Chan V, Fearon DF, Merad M, et al. Understanding the tumor immune microenvironment (TIME) for effective therapy. Nat Med. 2018;24(5):541–50. 10.1038/s41591-018-0014-x. PMC5998822 29686425

[37] Arneth B. Tumor microenvironment. Medicina (Kaunas). 2019;1:56. 10.3390/medicina56010015. PMC7023392 31906017

[38] Wang Y, Gu W, Wen W, Zhang X. SERPINH1 is a potential prognostic biomarker and correlated with immune infiltration: a pan-cancer analysis. Front Genet. 2021;12:756094. 10.3389/fgene.2021.756094. PMC8764125 35058967

[39] Rijavec E, Genova C, Biello F, Rossi G, Indini A, Grossi F. Current state of the art and future perspectives with immunotherapy in the management of small cell lung cancer. Expert Rev Respiratory Med. 2021;15(11):1427–35. 10.1080/17476348.2021.1987887. 34590937

