# Supplementary material for: Pan-cancer analysis of the PDE4DIP gene with potential prognostic and immunotherapeutic values in multiple cancers including acute myeloid leukemia
Source: Open Med (Wars). 2023 Aug 28;18(1):20230782. doi: 10.1515/med-2023-0782 (PMC10473463; doi:10.1515/med-2023-0782)
Supplement: Supplementary material [file med-2023-0782-sm.pdf]

Supplementary material

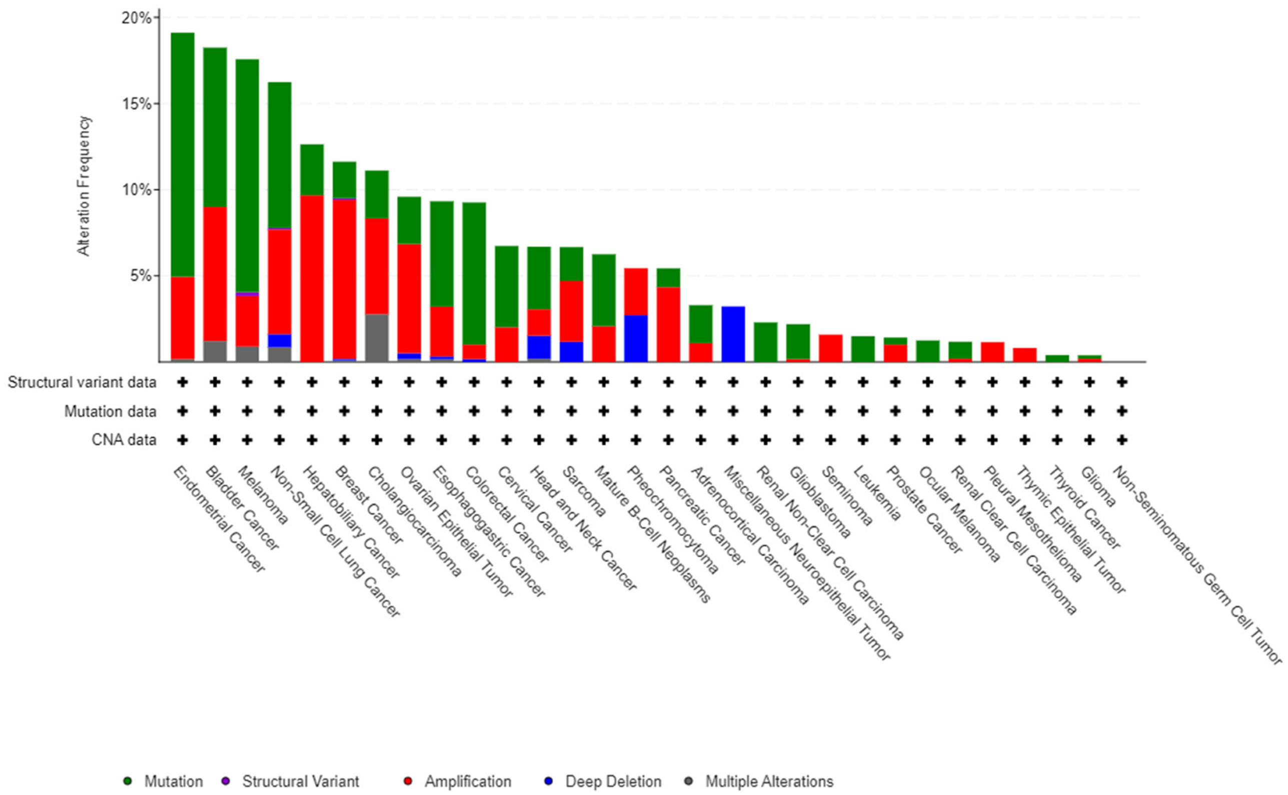

Figure S1: Association of the expression of *PDE4DIP* gene with genetic variation in pan-cancer.

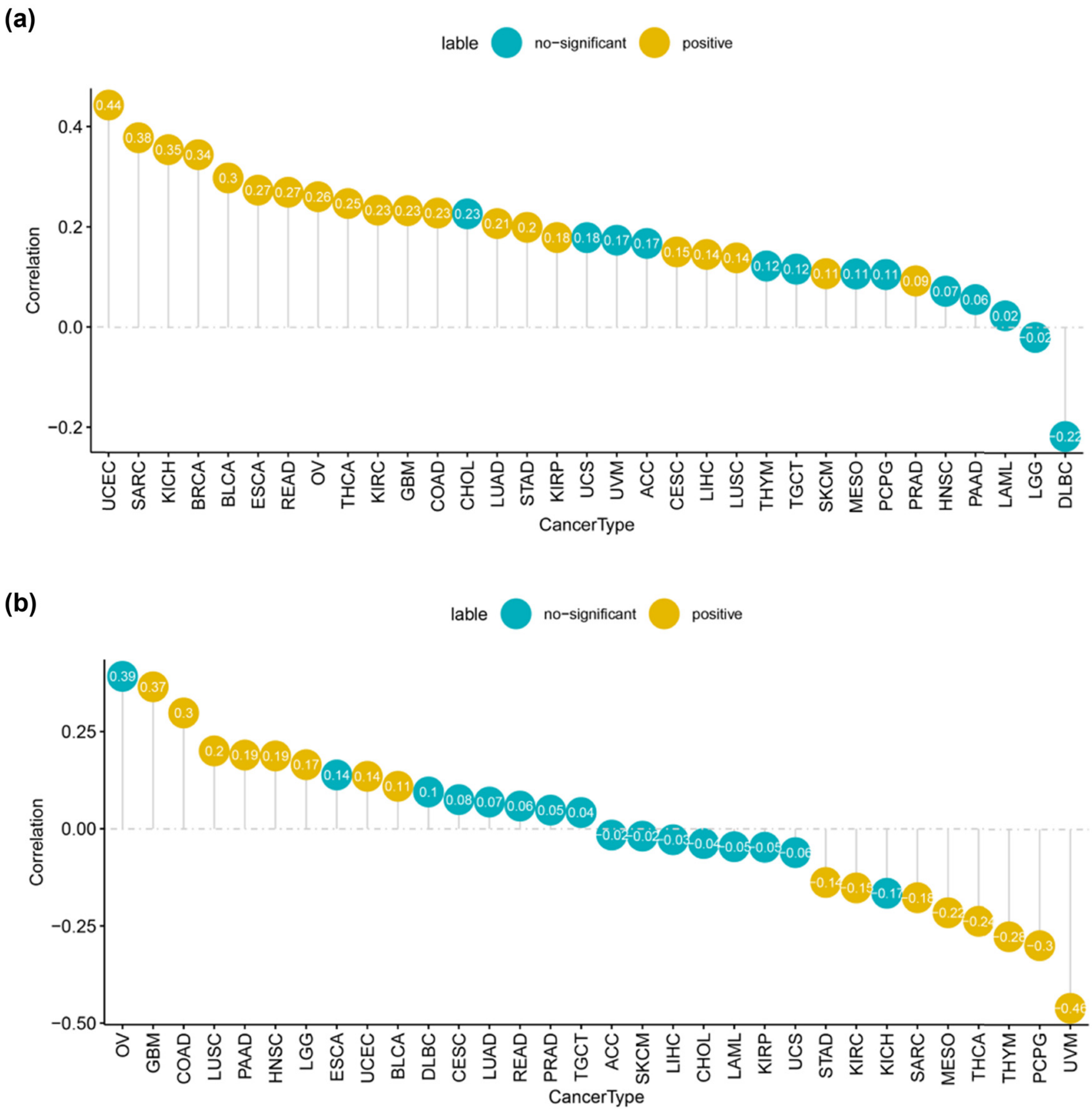

Figure S2: Association of *PDE4DIP* expression with DNA copy number (a) and DNA methylation (b) in pan-cancer.

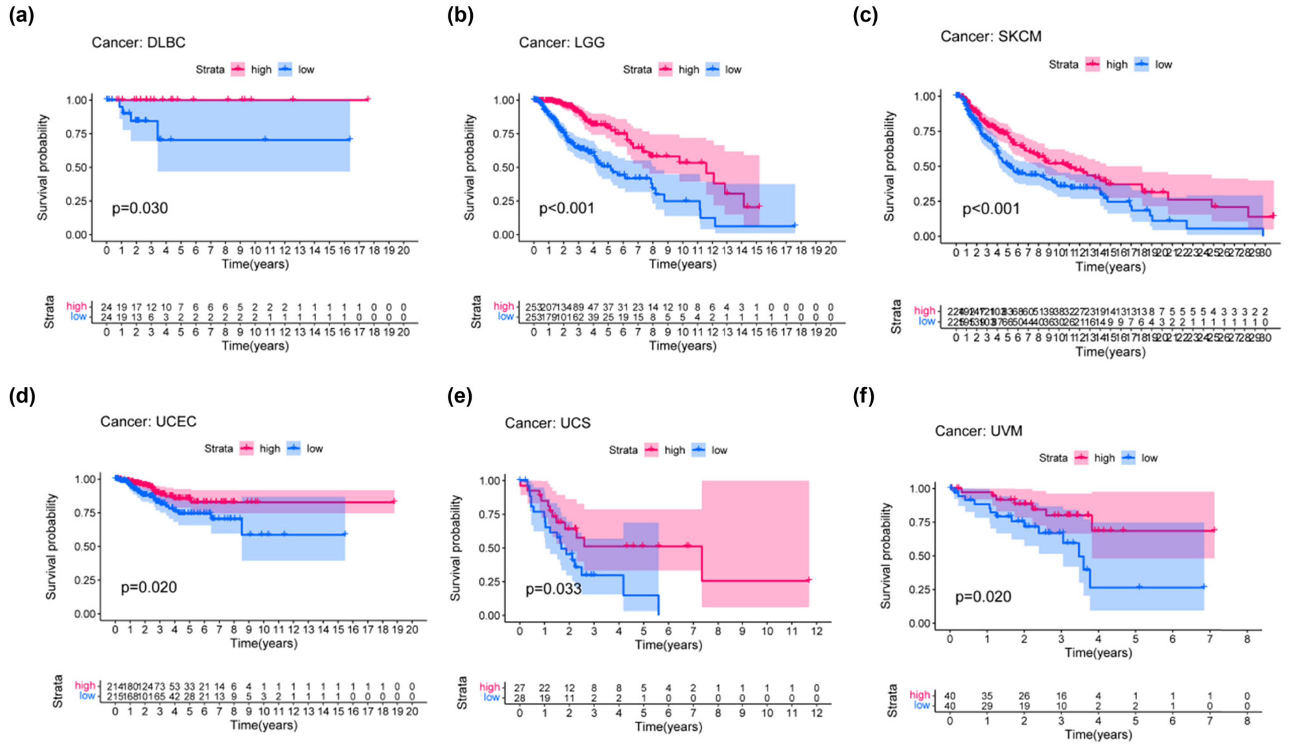

Figure S3: Association of *PDE4DIP* methylation and DSS in DLBC, LGG, SKCM, UCEC, UCS, and UVM. Show only significant results.

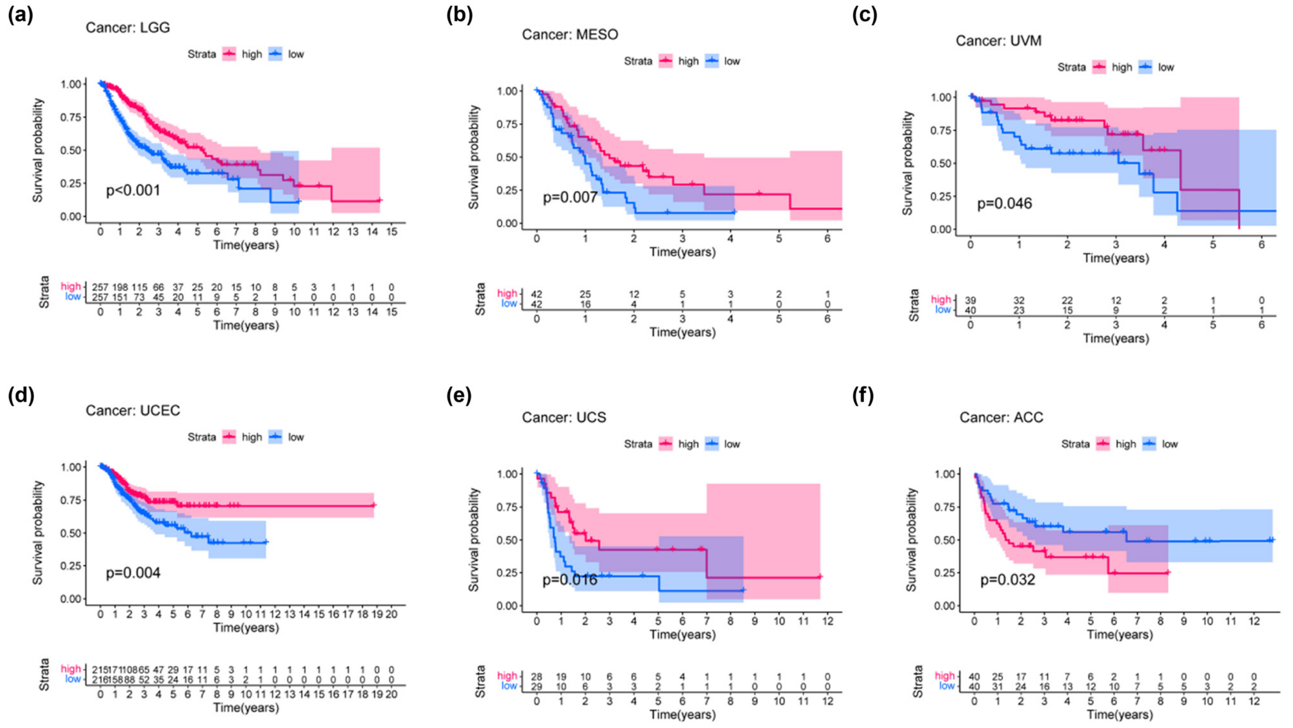

Figure S4: Association of *PDE4DIP* methylation and PFI in LGG, MESO, UVM, UCEC, UCS, and ACC. Show only significant results.

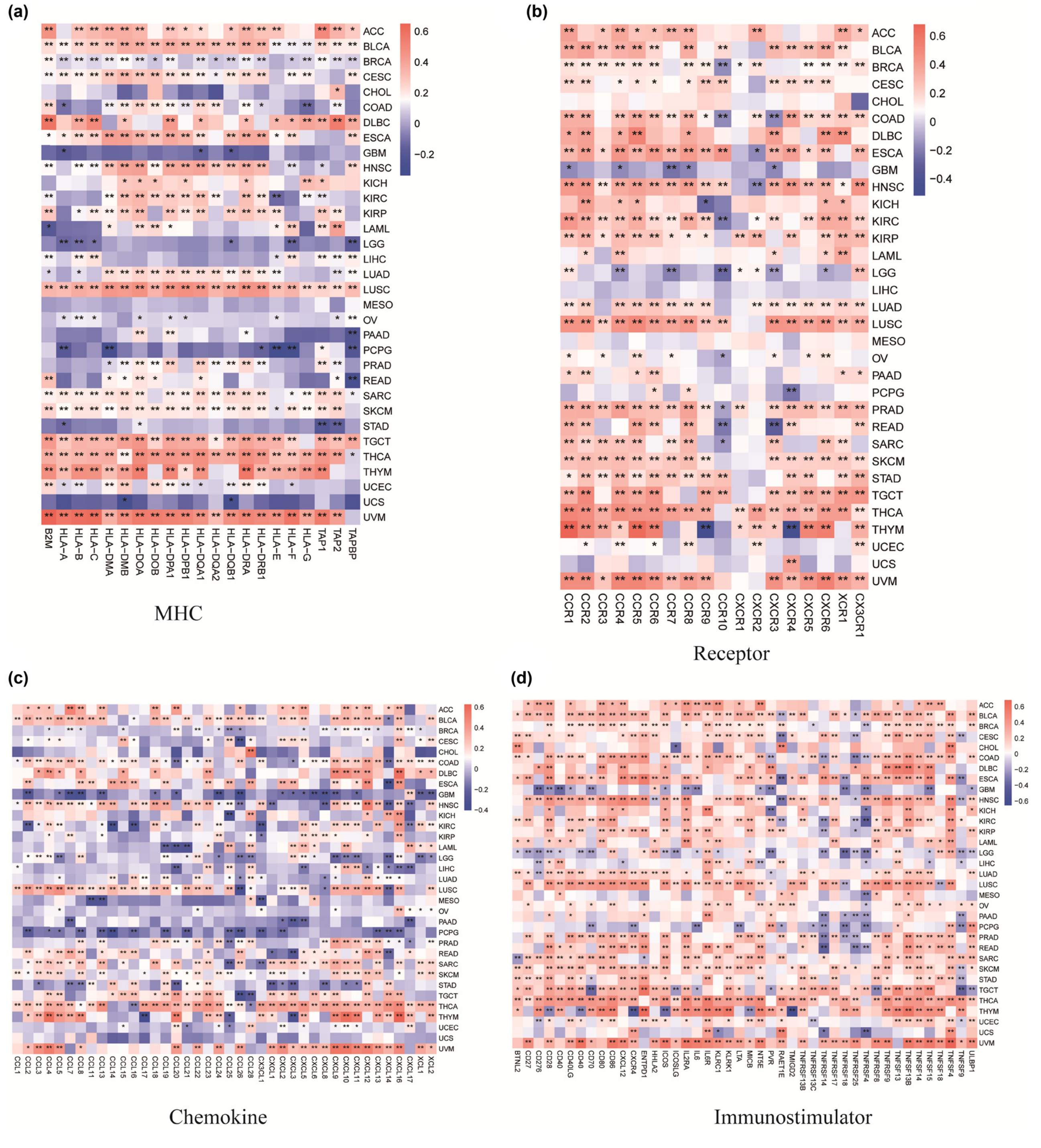

Figure S5: Correlation between *PDE4DIP* expression with MHC, receptor, chemokine in pan-cancer. *$p < 0.05$; **$p < 0.01$; and ***$p < 0.001$.

Table S1: The full names of 33 cancers come from TCGA database

Cancers	Full name
ACC	Adrenocortical carcinoma
BLCA	Bladder urothelial carcinoma
BRCA	Breast invasive carcinoma
CESC	Cervical squamous cell carcinoma and endocervical adenocarcinoma
CHOL	Cholangiocarcinoma
COAD	Colon adenocarcinoma
ESCA	Esophageal carcinoma
GBM	Glioblastoma multiforme
HNSC	Head and neck squamous cell carcinoma
KICH	Kidney chromophobe
KIRC	Kidney renal clear cell carcinoma
KIRP	Kidney renal papillary cell carcinoma
LAML	Acute myeloid leukemia
LGG	Brain lower grade glioma
LIHC	Liver hepatocellular carcinoma
LUAD	Lung adenocarcinoma
LUSC	Lung squamous cell carcinoma
OV	Ovarian serous cystadenocarcinoma
PAAD	Pancreatic adenocarcinoma
PCPG	Pheochromocytoma and Paraganglioma
PRAD	Prostate adenocarcinoma
READ	Rectum adenocarcinoma
SARC	Sarcoma
SKCM	Skin Cutaneous Melanoma
STAD	Stomach adenocarcinoma
TGCT	Testicular germ cell tumors
THCA	Thyroid carcinoma
THYM	Thymoma
UCEC	Uterine Corpus Endometrial Carcinoma
UCS	Uterine Carcinosarcoma
BLBC	Basal-like breast cancer
MESO	Mesothelioma
UVM	Uveal Melanoma

Table S2: Differential expression of *PDE4DIP* at mRNA level in tumor tissues and normal tissues

Cancers	Mean expression in tumor tissues Log2(FPKM+1)	Mean expression in normal tissues Log2(FPKM+1)	Log2FC	FC	*p*-value	Tumor vs Normal
ACC	2.57	1.69	0.88	1.83	4.16×10^{-14}***	up
BLCA	3.12	2.73	0.39	1.31	1.81×10^{-5}***	up
BRCA	3.7	3.34	0.35	1.28	2.60×10^{-21}***	up
CESC	2.98	3.03	−0.05	0.96	0.675	—
CHOL	3.2	3.1	0.10	1.07	0.571	—
COAD	2.84	2.93	−0.09	0.94	0.043	—
ESCA	3.03	3.17	−0.14	0.91	0.182	—
GBM	4.21	4.15	0.06	1.05	0.233	—
HNSC	3.05	3.68	−0.64	0.64	0.293	—
KICH	3.07	3.53	−0.46	0.73	0.005**	down
KIRC	3.4	3.85	−0.45	0.73	5.04×10^{-13}***	down
KIRP	3.38	3.51	−0.13	0.91	0.024*	down
LAML	2.56	5.68	−3.12	0.12	3.71×10^{-230}***	down
LGG	4.89	4.34	0.55	1.47	3.78×10^{-39}***	up
LIHC	3.36	2.89	0.47	1.38	4.11×10^{-19}***	up
LUAD	3.58	3.8	−0.23	0.85	1.32×10^{-13}***	down
LUSC	3.08	3.8	−0.72	0.61	1.29×10^{-97}***	down
OV	3.35	2.03	1.32	2.50	6.58×10^{-91}***	up
PAAD	3.32	2.21	1.10	2.15	5.27×10^{-41}***	up
PCPG	3.74	2.3	1.44	2.72	6.86×10^{-7}***	up
PRAD	3	2.81	0.19	1.14	1.55×10^{-5}***	up
READ	2.79	3.05	−0.26	0.84	0.018*	down
SARC	3.67	3.05	0.62	1.53	0.197	—
SKCM	3.16	3.54	−0.38	0.77	2.26×10^{-14}***	down
STAD	3.16	3.02	0.14	1.10	0.032*	up
TGCT	2.96	2.33	0.63	1.54	2.48×10^{-52}***	up
THCA	3.19	2.88	0.31	1.24	6.09×10^{-25}***	up
THYM	2.8	3.21	−0.41	0.75	0.205	—
UCEC	3.12	2.93	0.19	1.14	7.20×10^{-5}***	up
UCS	3.15	2.94	0.45	1.37	8.43×10^{-6}***	up

Note: *$p < 0.05$; **$p < 0.01$; and ***$p < 0.001$.

Table S3: The detailed results of the significance analysis of *PDE4DIP* protein expression level in pan-cancers from CPTAC dataset

CancerType	Comparison	*p*-value
Breast cancer (BRCA)	Normal-vs-Primary	4.60×10^{-16}*
Ovarian cancer (OV)	Normal-vs-Primary	6.92×10^{-1}
Colon cancer (COAD)	Normal-vs-Primary	2.05×10^{-3}*
Clear cell renal cell carcinoma (KIRC)	Normal-vs-Primary	2.15×10^{-13}*
Uterine corpus endometrial carcinoma (UCEC)	Normal-vs-Primary	7.25×10^{-29}*
Lung adenocarcinoma (LUAD)	Normal-vs-Primary	2.61×10^{-9}*
Head and neck squamous carcinoma (HNSC)	Normal-vs-Primary	5.98×10^{-3}*
Pancreatic adenocarcinoma (PAAD)	Normal-vs-Primary	3.39×10^{-6}*
Glioblastoma multiforme (GBM)	Normal-vs-Primary	1.31×10^{-3}*
Hepatocellular carcinoma (LIHC)	Normal-vs-Primary	3.33×10^{-3}*
Prostate adenocarcinoma (PRAD)	Age(41-60Yrs)-vs-Age(61-80Yrs)	9.39×10^{-1}
Gastric cancer (STAD)	Microsatellite instability-High-vs-Microsatellite instability-Low	5.81×10^{-1}

Note:*$p < 0.05$ indicates statistically significant.

Table S4: Comparison of *PDE4DIP* expression pattern at mRNA and protein level

Cancers	***PDE4DIP* mRNA expression (Tumor vs Normal)**	***PDE4DIP* protein expression (Tumor vs Normal)**
ACC	up-regulated	—
BLCA	up-regulated	—
BRCA	up-regulated	up-regulated
LGG	up-regulated	—
LIHC	up-regulated	up-regulated
OV	up-regulated	ns
PAAD	up-regulated	down-regulated
PCPG	up-regulated	—
PRAD	up-regulated	ns
STAD	up-regulated	ns
TGCT	up-regulated	—
THCA	up-regulated	—
UCEC	up-regulated	up-regulated
UCS	up-regulated	–
KICH	down-regulated	–
KIRC	down-regulated	down-regulated
KIRP	down-regulated	—
LAML	down-regulated	—
LUAD	down-regulated	up-regulated
LUSC	down-regulated	—
READ	down-regulated	—
SKCM	down-regulated	—
COAD	ns	down-regulated
GBM	ns	up-regulated
HNSC	ns	up-regulated
CESC	ns	—
CHOL	ns	—
SARC	ns	—
THYM	ns	—
ESCA	ns	—

Note: ns indicates no statistical significance.
